# A Giant Choledochal Cyst Mimicking Pancreatic Pseudocyst: A Case Report

**DOI:** 10.7759/cureus.29806

**Published:** 2022-10-01

**Authors:** Fathia Harrabi, Houssem Ammar, Mahdi Ben Latifa, Mohamed Amine Said, Raoudha Chrigui, Habiba Ben Hamada, Rahul Gupta, Mohamed Ben Mabrouk, Ali Ben Ali

**Affiliations:** 1 Gastrointestinal Surgery, Sahloul Hospital, University of Sousse, Sousse, TUN; 2 Anesthesiology and Critical Care, Sahloul Hospital, University of Sousse, Sousse, TUN; 3 Gastrointestinal Surgery, Synergy Institute of Medical Sciences, Dehradun, IND

**Keywords:** general surgery, magnetic resonance cholangiopancreatography, acute pancreatitis, pancreatic pseudocyst, choledochal cyst

## Abstract

A choledochal cyst (CC) is a rare congenital anomaly manifested as cystic dilatation of the biliary tree. A giant choledochal cyst is defined as a cyst with a maximum diameter of ≥ 10 cm. It is an unusual entity and rarely revealed in adulthood. We describe the case of a giant, infected CC presenting as acute pancreatitis with results of abdominal ultrasound and computed tomography consistent with a pancreatic pseudocyst. The diagnosis of CC was made on magnetic resonance cholangiopancreatography (MRCP) findings. We proceeded with cholecystectomy and the complete resection of the diverticulum after its dissection. The defect in the common bile duct was closed transversally over a T-tube. At the last follow-up two years after his admission, the patient is symptom-free with normal liver enzymes

To the best of our knowledge, this is the first case reported of giant CC complicated with both infection and pancreatitis

## Introduction

A choledochal cyst (CC) is a rare congenital anomaly manifested as cystic dilatation of the biliary tree. A giant choledochal cyst is defined as a cyst with a maximum diameter of ≥ 10 cm. It is an unusual entity and rarely revealed in adulthood [1]. From 1977, the Todani classification has become the basic system of classification of choledochal cysts. Type II CC is very rare (2-3%) and appears as an extrahepatic, supraduodenal diverticulum [2].

In adults, choledochal cysts are mostly asymptomatic. Rarely, biliary perforation, hepatic abscess, biliary cirrhosis, and acute pancreatitis may be the first presentation [3]. Small CC can be easily diagnosed with radiology. However, a large CC can compress the adjoining organs such as the liver, head of the pancreas, duodenum and right kidney and appear to arise from these organs. However, accurate preoperative diagnosis is important to manage such large cysts.

We describe the case of giant, infected CC presenting as acute pancreatitis and misdiagnosed as a pancreatic pseudocyst. To the best of our knowledge, this is the first case reported in the literature, and surgeons should consider this pathology while treating pancreatic pseudocysts.

## Case presentation

A 43-year-old man presented with severe abdominal pain, nausea, and vomiting for four days. The pain was dull aching in nature, paroxysmal, and unrelated to food and without jaundice. The patient has had episodic epigastric and right upper quadrant abdominal pain for the last two years, which was not explored. He was a current smoker with regular consumption of alcohol. On physical examination, he was febrile (temperature of 38.5 °Celsius), had tachycardia, and had tenderness in the epigastric and right upper quadrant. Laboratory investigations revealed leukocytosis and elevated pancreatic enzymes.

**Figure 1:**
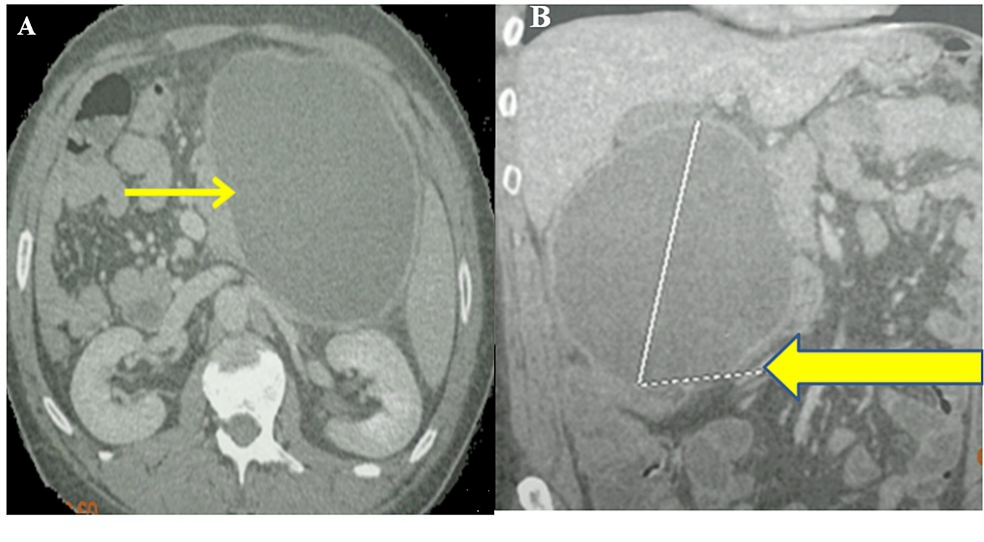
Contrast-enhanced computed tomography showing a round, well-circumscribed fluid collection of 17 x 10 cm (yellow arrow) on axial (A) and coronal sections (B).

Contrast-enhanced computed tomography of the abdomen (CT) showed a round, well-circumscribed fluid collection of 17 x 10 cm with a thick enhancing wall suggestive of pancreatic pseudocyst with interstitial edematous pancreatitis (Figure 1).

**Figure 2:**
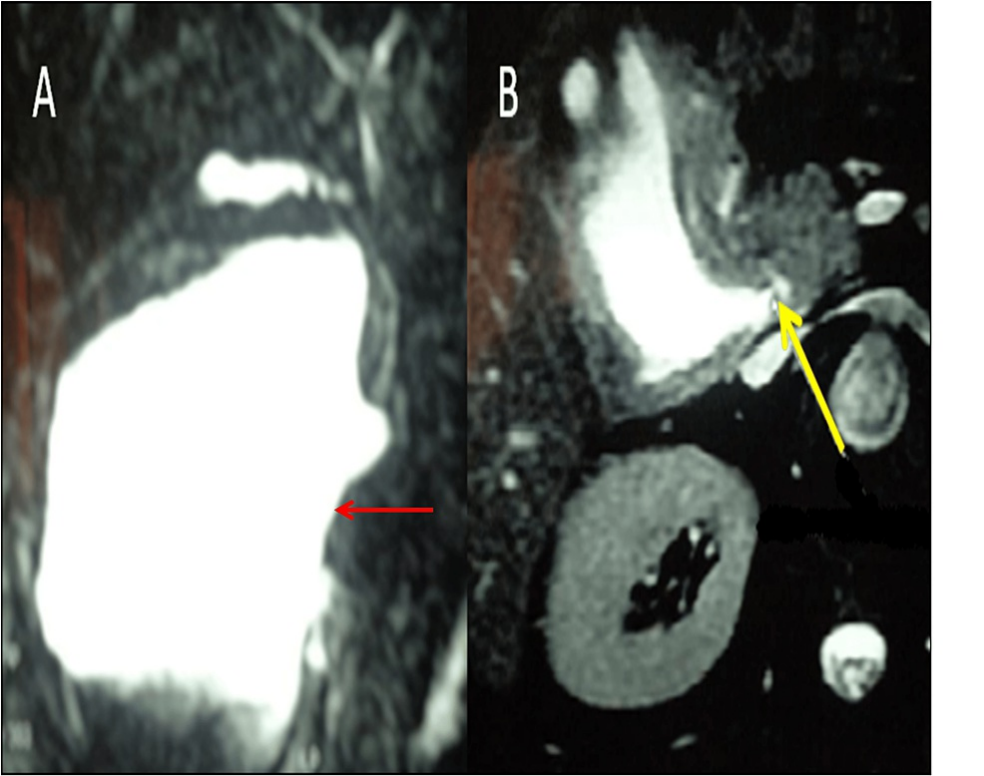
Magnetic resonance cholangiopancreatography showing a giant cystic formation of 20 x 16 cm arising from the supraduodenal extrahepatic bile duct (A) and the defect in the common bile duct (yellow arrow) (B).

Based on the clinical and imaging findings, a provisional diagnosis of infected pancreatic pseudocyst was made. Broad-spectrum antibiotics and fluid resuscitation were started immediately and CT-guided percutaneous drainage of this collection was performed, bringing 2 liters of infected bile colonized by *Staphylococcus aureus *on bacteriological examination. Due to the presence of bile in the drainage fluid, we performed a magnetic resonance cholangiopancreatography (MRCP), which revealed a giant cyst of 20 x 16 cm arising from the supraduodenal extrahepatic bile duct without dilatation of extra and intrahepatic ducts suggestive of Type II CC as per Todani classification (Figure 2).

**Figure 3:**
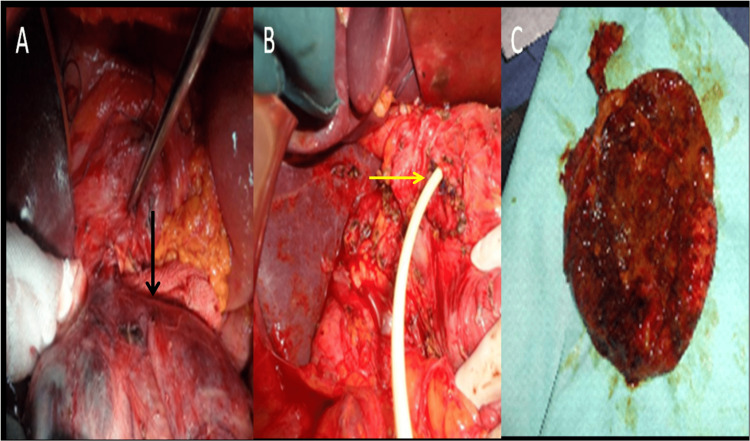
Intra-operative images showing saccular diverticulum in the right upper quadrant (black arrow) (A); the defect in the common bile closed over a T-tube (yellow arrow) (B); and the specimen of the excised choledochal cyst (C).

Intra-operatively, the diverticulum was located at the middle part of the extrahepatic biliary tree pushing the duodenum and the pancreas. We proceeded with cholecystectomy and the complete resection of the diverticulum after its dissection. The defect in the common bile duct was closed transversally over a T-tube (Figure 3).

The excised choledochal cyst was sent for histopathological examination and showed no evidence of malignancy. The patient was discharged after five days of hospitalization. A postoperative cholangiography showed a non-dilated common bile duct, no stones, and normal passage to the duodenum. At the last follow-up two years after his admission, the patient is symptom-free with normal liver enzymes and tumor markers.

## Discussion

Todani type II choledochal cysts are very rare and account for 2-3% of all bile duct cysts. Also known as a bile duct diverticulum, saccular cysts represent a true diverticulum arising from the supraduodenal extrahepatic bile duct. They represent a predominantly pediatric pathology, but in 20% of cases, they can also be found in adults, with a higher incidence in females [3-6]. In adults, choledochal cysts are rarely symptomatic and incidentally discovered [4].

Most of the giant CCs are of type IVA and type I, according to Todani’s classification. The symptoms are more severe for giant CCs compared to non-giant CCs, and the classical triad of pain, abdominal mass, and jaundice was present in the majority of the patients with giant CCs [1,5]. Several cases of giant cysts (over 10 cm) were reported in the literature, mostly discovered in the context of a complication linked to their significant size [3, 5, 7].

Differential diagnosis of CC includes intraabdominal cystic lesion of the mesentery, peritoneum, pancreas or gastrointestinal tract [1,8]. CC can be misdiagnosed as a pancreatic cystic lesion or duodenal duplication cyst. Acute pancreatitis is rarely reported as a presentation of giant CCs in adults. Giant CCs mimicking a pancreatic pseudocyst and having complications of both infection and acute pancreatitis have not yet been reported. MRCP and the ERCP (endoscopic retrograde cholangiopancreaticography) are the most sensitive imaging studies for the diagnosis of CCs [1, 3, 5].

Surgery is the treatment choice for CCs to avoid the risk of complications such as pancreatitis, choledocholithiasis, or malignant degeneration. The surgical treatment depends on Todani classification and includes complete cyst excision (including gallbladder) with biliary-enteric anastomosis if the common bile duct is affected [1, 2,5]. The surgical procedure involves complete resection of the lesion, cholecystectomy, choledochorraphy and without biliary-enteric reconstruction. Traditionally, laparotomy is the common surgical approach to giant choledochal cysts. However, laparoscopic surgery has successful results, especially for pediatric patients [1, 6,9].

## Conclusions

Giant CC is a very rare entity in the spectrum of congenital bile duct abnormality with few cases reported in the literature. The diagnosis requires high clinical suspicion and should be included in the differential diagnosis while treating a cystic pancreatic lesion. Also, to the best of our knowledge, this is the first case reported of giant CC complicated with both infection and pancreatitis.
